# Sperm Selection for micro TESE-ICSI in Non-Obstructive Azoospermia, a Case Report

**DOI:** 10.5935/1518-0557.20210012

**Published:** 2021

**Authors:** Mauro Bibancos, Rodrigo Markus Vaz, Paulo Ferreira Mega, Edson Borges Jr., Mariana Ribeiro, Davi Buttros, Wagner Eduardo Matheus, Ilaria Cosci, Carlo Foresta, Andrea Garolla

**Affiliations:** 1 Pontifical Catholic University of Campinas, Brazil; 2 Fivmed Laboratory for Human Reproduction, Campinas, Brazil; 3 Fertility Medical Group, São Paulo, Brazil; 4 State University of Campinas, Campinas, Brazil; 5 Unit of Andrology and Reproductive Medicine & Centre for Male Gamete Cryopreservation, Department of Medicine, University of Padova, Padova, Italy

**Keywords:** ART failure, birefringence, high magnification microscopy, *in-vitro* fertilization, non-obstructive azoospermia, sperm selection

## Abstract

TESE-ICSI (testicular sperm extraction associated with intracytoplasmic sperm injection) represents a technique to attain pregnancy in couples with non-obstructive azoospermia (NOA) and other unlikely situations. Because of the poor pregnancy outcomes obtained by this procedure, we need new sperm selection techniques to improve the livebirth rate of NOA patients. Here we describe a successful micro TESE-ICSI cycle performed with sperm selected through high magnification and polarized light microscopy in a couple with two previous ICSI failures.

## INTRODUCTION

Non-obstructive azoospermia (NOA) is considered a non-medically treatable cause of male infertility ^(Flannigan *et al*., 2017)^. NOA patients constitute up to 10% of all infertile men, and have abnormal spermatogenesis as the cause of their azoospermia ^(Flannigan *et al*., 2017)^. In this condition, standard techniques to retrieve sperm are testicular sperm extraction (TESE) and testicular sperm extraction with micro dissection (micro TESE), having a reported success rate of 55% ^(Flannigan *et al*., 2017)^ and 63%, respectively ^(Donoso *et al*., 2007; Shah & Gupta, 2017)^. In case of successful extraction, the standard procedure is to analyze the retrieved spermatozoa under a 40x magnification microscope, with the purpose of selecting the ones with the most adequate morphology and motility to use for intracytoplasmic sperm injection (ICSI).

Due to severe testicular failure, sperm from NOA patients have higher percentages of aneuploidy, altered DNA compaction, and both single and double stranded DNA breakages ^(Arumugam *et al*., 2019; Garolla *et al*., 2017)^. This condition is believed to affect assisted reproduction outcome ^(Garolla *et al*., 2015)^, and many authors report a strong association between poor semen quality and lower cumulative live birth rates ^(Kamkar *et al*., 2018; Zacà *et al*., 2020)^. On this basis, there are advanced sperm selection techniques, proposed to improve the likelihood that chromosomally intact and mature sperm with high DNA integrity are selected for fertilization ^(Pérez-Cerezales *et al*., 2018; Simopoulou *et al*., 2016)^. These strategies include selection according to membrane charge, apoptosis, birefringence, thermotaxis, chemotaxis and normal morphology, observed at higher magnification. Although the current evidence is insufficient to reach final conclusions, these techniques could theoretically improve outcomes in assisted reproduction ^(Lepine *et al*., 2019)^. However, there is no method suggested for the selection of sperm retrieved by TESE. The aim of this case report is to report on the fertility outcome in a couple with NOA, undergoing the third ICSI cycle, in which sperm were retrieved by micro TESE and selected by birefringence through polarized light under high magnification microscopy.

## CASE REPORT

A couple with male factor infertility underwent two unsuccessful micro-TESE-ICSI cycles. The female partner was 37 years old, body mass index (BMI) of 22, no history of previous surgeries or use of medications and regular menstrual cycles. The male partner was 37 years old, with idiopathic NOA due to testicular failure, follicle stimulating hormone 11.3 IU/L, luteinizing hormone 8.3 IU/L, testicular volume 12mL bilaterally and no history of orchidopexy, orchitis, varicocele or hormonal treatments. We ran ICSI cycles with sperm retrieved by micro-TESE procedure. In the first cycle, we obtained 14 oocytes, 10 were MII and 6 were injected with non-progressive motile sperm, and 4 had non-motile but viable sperm, evaluated by the hyposmotic swelling test ^(Rossato *et al*., 2004)^. Upon day 3, three embryos developed only from oocytes injected with motile sperm. Thereafter, two embryos were transferred on the same day while the third stopped its development after 5 days of extended culture. We had no pregnancies. In the second cycle, we used sperm from the same micro-TESE procedure. We collected sixteen oocytes, all resulted in MII. We injected ten with non-progressive motile sperm and six with immotile, but viable sperm. We obtained the development of three embryos that were cryopreserved on the third day. In a later cycle, the embryos were warmed and cultured until the fifth day. We transferred one blastocyst, also with no pregnancy.

On a third ICSI cycle; we selected sperm obtained by the same micro-TESE procedure, for ICSI, using criteria based on high magnification morphology and birefringence, according to the protocol proposed by ^Garolla *et al*. (2014)^.

### Ovarian stimulation, oocyte fertilization and embryo culture

Controlled ovarian stimulation was the same in all ICSI cycles and happened as follows: pituitary blockade was achieved with a GnRH antagonist, and ovarian stimulation was performed with recombinant FSH initiated on day 3 of the menstrual cycle. The initial gonadotrophin dose was 100 UI and adjusted according to the ovarian response to 50UI + 1 ampule of Pergoveris^®^. We monitored follicle development using ultrasound; when at least two follicles were ≥18 mm in diameter, we triggered final oocyte maturation with Ovidrel^®^. Oocyte aspiration was performed 35 h after triggering. The oocytes were denuded and then assessed for maturity stage. All mature metaphase II (MII) oocytes were fertilized by intracytoplasmic sperm injection (ICSI) ^(Palermo *et al*., 1992)^. On day 1 (D1), the normally fertilized oocytes - defined as having two pronuclei (2PN) and two polar bodies - were identified and cultured in microdroplets from day 1 until blastocyst stage (D5 or D6) 50 µL of medium containing 10% human albumin (CSCM, Irvine Scientific) under 11 mL of paraffin oil. The embryos were then incubated in triple gas incubators (89% N_2_, 5% O_2_ and 6% CO_2_). The blastocysts were morphologically classified according to ^Gardner *et al*. (2000)^. The cycles with blastocyst formation underwent fresh transfer or blastocyst cryopreservation.

### Micro-TESE

We performed the Micro-TESE under epidural sedation in a hospital. The procedure was handled with microsurgical material and under light microscopy, with 6-8x magnification. The surgeon provided areas of testicles to be removed. During the procedure, all removed seminiferous tubules, were analyzed by an andrologist in the surgical room. The sample was flushed into a sterile *Petri* dish, containing culture medium: Multipurpose Handling Media (MHM) supplemented with 5% HSA (Irvine Scientific, Santa Ana, CA, USA). We used BD PrecisionGlide^TM^ 0.80X30 mm (21GX11/4") sterile and disposable needles to dissect the seminiferous tubules. We examined an aliquot of this fluid under the light microscope with phase contrast and objective magnification of 20X, to ascertain the presence of spermatozoa, on microscope slides covered with glass. The samples were placed into a conical tube with mHTF, plus 10% SSS and transported to laboratory at 37°C. We centrifuged the sample at 1200 rpm and transferred the pellet to a *Petri* dish with Multipurpose Handling Media (MHM), supplemented with 5% HSA covered with mineral oil. After sperm identification, sperm aliquots to be frozen were diluted with human sperm cryopreservation medium (Irvine Scientific, Santa Ana, CA, USA). The diluted aliquots were drawn into 0.5 ml sterile plastic straws (CryoBiosystem, L'Aigle, France) and cryopreserved as follows: the protocol used 0.5 mL straws and the freezing was conducted in LN steam for 3 min, 3 cm over the LN surface, before immersion and storage at -196°C. For the thawing procedure, the straws were placed upright on a rack at room temperature until all visible ice was gone.

### Sperm selection for ICSI by high magnification microscopy and birefringence

After thawing, the sample was washed by centrifugation at 300 g for 10 minutes. The pellet was incubated in 10-µL microdrops of PVP 7% (polyvinylpyrrolidone solution; Origio Medicult Media, Måløv, Denmark) in a Petri dish, covered with liquid paraffin (Origio Medicult Media) at room temperature for 15 minutes, and immediately used for further analysis. Motile sperm were evaluated under high magnification for morphology and birefringence, as previously reported ^(Garolla *et al*., 2014)^. In brief, each sperm was assessed using an inverted microscope (Eclipse TE 2000 U; Nikon) equipped with high-power differential interference contrast optics (Nomarski), with Hoffman contrast and polarizing lenses. The morphology evaluation criteria were defined according to ^Bartoov *et al*. (2002)^, observing sperm at higher magnification, as previously reported ^(Garolla *et al*., 2008)^. The combined analysis took approximately 5 minutes per sperm. Only birefringent motile sperm with normal morphology and no vacuoles were selected and used for ICSI.

## RESULTS

**Figure 1:**
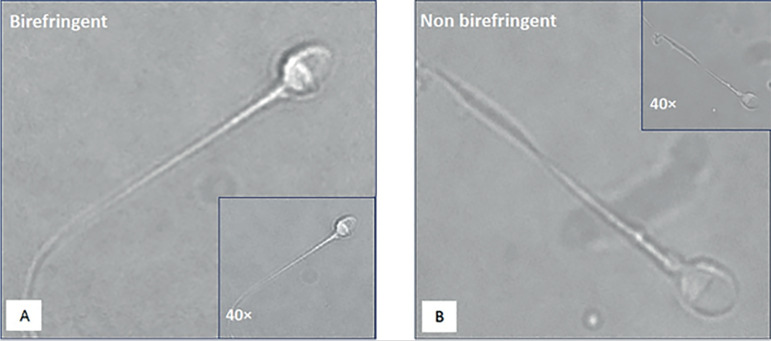
Two examples of spermatozoa retrieved by micro TESE, selected at high magnification, and polarized light microscopy. In the small windows, the same cells seen upon standard magnification. A) Birefringent sperm with non-progressive motility and normal morphology. B) Non-birefringent sperm with non-progressive motility and normal morphology.

After two previously failed ICSI cycles, we ran a new attempt with the same stimulation protocol and using sperm obtained by the same micro-TESE procedure. In this cycle, the sperm were selected by using criteria based on high magnification morphology and birefringence, according to the protocol previously proposed by ^Garolla *et al*. (2014)^. In Figure 1 there are two examples of spermatozoa with non-progressive motility and normal morphology, retrieved by micro TESE, selected under high magnification, and polarized light microscopy. Pictures A and B show a birefringent and a non-birefringent sperm, respectively. In the third ICSI cycle, ten MII oocytes were collected, and injected with non-progressive motile, birefringent sperm with normal morphology. Seven fertilized embryos were obtained, five of which reached the blastocyst stage on days 5 and 6 reached embryonic development. After NGS analysis, all embryos were diagnosed as euploid. In a subsequent cycle, an euploid embryo was warmed, transferred and after 12 days the β-hCG resulted positive.

## DISCUSSION

Infertile men, particularly those with NOA, have higher percentages of sperm aneuploidy and DNA fragmentation due to abnormal spermatogenesis ^(Calogero *et al*., 2003; Flannigan *et al*., 2017)^. Therefore, couples with NOA frequently face in-vitro fertilization failures, including poor fertilization, reduced implantation or increased miscarriage rates ^(Alvarez Sedó *et al*., 2017; Calogero *et al*., 2003)^. In these couples the conventional technique used to retrieve sperm is represented by TESE ^(Donoso *et al*., 2007)^. Recently micro-TESE, performed under a microscopic observation, has been suggested as a more effective method of sperm retrieval because of its capability to identify areas with higher likelihood of having spermatozoa ^(Wosnitzer *et al*., 2014)^. However, the problem regarding the quality of the sperm retrieved from an impaired testicle remains ^(Loutradi *et al*., 2006)^. On this basis, there are concerns regarding the use of intratesticular sperm from NOA patients for assisted reproduction ^(Loutradi *et al*., 2006)^. These cells are frequently non-mobile, thus it is difficult to understand their viability. Moreover, the lack of motility has been related to changes to the intermediate piece (centriole), that is a preponderant factor for the beginning of embryonic cleavage ^(Tesarik, 2005)^. In this context, embryologists select motile sperm, when present, in order to improve ovular division and the cleavage process.

In recent years, many tests were developed to select sperm with reduced nuclear abnormalities from semen samples of infertile men candidate to assisted reproduction ^(Lepine *et al*., 2019; Mazzilli *et al*., 2017)^. Among these, birefringence associated with high magnification microscopy was reported as being able to select sperm with higher likelihood of having normal DNA ^(Garolla *et al*., 2014)^. Therefore, it has been suggested as a real-time analysis to select good-quality sperm, to improve the ICSI outcome for men with testicular damage ^(Garolla *et al*., 2014)^. Despite many papers reporting the selection of the best sperm for infertile men in couples with repeated ICSI failures, there is some data regarding this approach after testicular sperm extraction ^(Verheyen *et al*., 2017)^.

In this report, we describe the success of a micro TESE-ICSI procedure, performed for the first time with a particular technique of sperm selection, in a couple who experienced two previous treatment failures. Non-progressive motile sperm were selected at high magnification microscopy with a polarized light microscope, to obtain morphologically normal and birefringent sperm. From ten MII oocytes injected with non-progressive motile and birefringent sperm with normal morphology, five euploid blastocysts were obtained. The transfer of an euploid embryo, performed in a subsequent cycle, allowed the couple to get pregnant.

Even though this event could be attributed to a role of chance, this technique represents a new approach for couples with non-obstructive azoospermia. It is worth stressing that in all three attempts the ovulation induction was the same and the retrieved sperm derived from the same micro TESE procedure. The only difference was the use of high magnification microscopy and birefringence for sperm selection in the third attempt.

In conclusion, we speculate that implementing the sperm selection after micro TESE, using high magnification morphology and birefringence, the analysis could be much deeper, providing better ICSI outcomes. Despite our results, it is clear that larger studies should be performed to assess the real benefits of this technique, helping to analyze whether or not it could become a new option for NOA patients undergoing ICSI.

## References

[r1] Alvarez Sedó C, Bilinski M, Lorenzi D, Uriondo H, Noblía F, Longobucco V, Lagar EV, Nodar F (2017). Effect of sperm DNA fragmentation on embryo development: clinical and biological aspects. JBRA Assist Reprod.

[r2] Arumugam M, Shetty DP, Kadandale JS, Nalilu SK (2019). Association of Sperm Aneuploidy Frequency and DNA Fragmentation Index in Infertile Men. J Reprod Infertil.

[r3] Bartoov B, Berkovitz A, Eltes F, Kogosowski A, Menezo Y, Barak Y (2002). Real-time fine morphology of motile human sperm cells is associated with IVF-ICSI outcome. J Androl.

[r4] Calogero AE, Burrello N, De Palma A, Barone N, D'Agata R, Vicari E (2003). Sperm aneuploidy in infertile men. Reprod Biomed Online.

[r5] Donoso P, Tournaye H, Devroey P (2007). Which is the best sperm retrieval technique for non-obstructive azoospermia? A systematic review. Hum Reprod Update.

[r6] Flannigan R, Bach PV, Schlegel PN (2017). Microdissection testicular sperm extraction. Transl Androl Urol.

[r7] Gardner DK, Lane M, Stevens J, Schlenker T, Schoolcraft WB (2000). Blastocyst score affects implantation and pregnancy outcome: towards a single blastocyst transfer. Fertil Steril.

[r8] Garolla A, Fortini D, Menegazzo M, De Toni L, Nicoletti V, Moretti A, Selice R, Engl B, Foresta C (2008). High-power microscopy for selecting spermatozoa for ICSI by physiological status. Reprod Biomed Online.

[r9] Garolla A, Cosci I, Menegazzo M, De Palo R, Ambrosini G, Sartini B, Pizzol D, Foresta C (2014). Sperm selected by both birefringence and motile sperm organelle morphology examination have reduced deoxyribonucleic acid fragmentation. Fertil Steril.

[r10] Garolla A, Cosci I, Bertoldo A, Sartini B, Boudjema E, Foresta C (2015). DNA double strand breaks in human spermatozoa can be predictive for assisted reproductive outcome. Reprod Biomed Online.

[r11] Garolla A, Ghezzi M, Cosci I, Sartini B, Bottacin A, Engl B, Di Nisio A, Foresta C (2017). FSH treatment in infertile males candidate to assisted reproduction improved sperm DNA fragmentation and pregnancy rate. Endocrine.

[r12] Kamkar N, Ramezanali F, Sabbaghian M (2018). The relationship between sperm DNA fragmentation, free radicals and antioxidant capacity with idiopathic repeated pregnancy loss. Reprod Biol.

[r13] Lepine S, McDowell S, Searle LM, Kroon B, Glujovsky D, Yazdani A (2019). Advanced sperm selection techniques for assisted reproduction. Cochrane Database Syst Rev.

[r14] Loutradi KE, Tarlatzis BC, Goulis DG, Zepiridis L, Pagou T, Chatziioannou E, Grimbizis GF, Papadimas I, Bontis I (2006). The effects of sperm quality on embryo development after intracytoplasmic sperm injection. J Assist Reprod Genet.

[r15] Mazzilli R, Cimadomo D, Vaiarelli A, Capalbo A, Dovere L, Alviggi E, Dusi L, Foresta C, Lombardo F, Lenzi A, Tournaye H, Alviggi C, Rienzi L, Ubaldi FM (2017). Effect of the male factor on the clinical outcome of intracytoplasmic sperm injection combined with preimplantation aneuploidy testing: observational longitudinal cohort study of 1,219 consecutive cycles. Fertil Steril.

[r16] Palermo G, Joris H, Devroey P, Van Steirteghem AC (1992). Pregnancies after intracytoplasmic injection of single spermatozoon into an oocyte. Lancet.

[r17] Pérez-Cerezales S, Laguna-Barraza R, de Castro AC, Sánchez-Calabuig MJ, Cano-Oliva E, de Castro-Pita FJ, Montoro-Buils L, Pericuesta E, Fernández-González R, Gutiérrez-Adán A (2018). Sperm selection by thermotaxis improves ICSI outcome in mice. Sci Rep.

[r18] Rossato M, Galeazzi C, Ferigo M, Foresta C (2004). Antisperm antibodies modify plasma membrane functional integrity and inhibit osmosensitive calcium influx in human sperm. Hum Reprod.

[r19] Shah R, Gupta C (2017). Advances in sperm retrieval techniques in azoospermic men: A systematic review. Arab J Urol.

[r20] Simopoulou M, Gkoles L, Bakas P, Giannelou P, Kalampokas T, Pantos K, Koutsilieris M (2016). Improving ICSI: A review from the spermatozoon perspective. Syst Biol Reprod Med.

[r21] Tesarik J (2005). Paternal effects on cell division in the human preimplantation embryo. Reprod Biomed Online.

[r22] Verheyen G, Popovic-Todorovic B, Tournaye H (2017). Processing and selection of surgically-retrieved sperm for ICSI: a review. Basic Clin Androl.

[r23] Wosnitzer M, Goldstein M, Hardy MP (2014). Review of Azoospermia. Spermatogenesis.

[r24] Zacà C, Coticchio G, Tarozzi N, Nadalini M, Lagalla C, Garolla A, Borini A (2020). Sperm count affects cumulative birth rate of assisted reproduction cycles in relation to ovarian response. J Assist Reprod Genet.

